# Deep learning-based six-type classifier for lung cancer and mimics from histopathological whole slide images: a retrospective study

**DOI:** 10.1186/s12916-021-01953-2

**Published:** 2021-03-29

**Authors:** Huan Yang, Lili Chen, Zhiqiang Cheng, Minglei Yang, Jianbo Wang, Chenghao Lin, Yuefeng Wang, Leilei Huang, Yangshan Chen, Sui Peng, Zunfu Ke, Weizhong Li

**Affiliations:** 1 Zhongshan School of Medicine, Sun Yat-sen University, Guangzhou, 510080 China; 2 Department of Pathology, The First Affiliated Hospital, Sun Yat-sen University, Guangzhou, 510080 China; 3 Department of Pathology, Shenzhen People’s Hospital, Shenzhen, 518020 China; 4 Center for Precision Medicine, Sun Yat-sen University, Guangzhou, 510080 China; 5 Molecular Diagnosis Center or Institute of Precision Medicine, The First Affiliated Hospital, Sun Yat-sen University, Guangzhou, 510080 China; 6 Key Laboratory of Tropical Disease Control (Ministry of Education), Sun Yat-sen University, Guangzhou, 510080 China

**Keywords:** Deep learning, Lung cancer, Cancer mimic, Whole slide image, Histopathological classification

## Abstract

**Background:**

Targeted therapy and immunotherapy put forward higher demands for accurate lung cancer classification, as well as benign versus malignant disease discrimination. Digital whole slide images (WSIs) witnessed the transition from traditional histopathology to computational approaches, arousing a hype of deep learning methods for histopathological analysis. We aimed at exploring the potential of deep learning models in the identification of lung cancer subtypes and cancer mimics from WSIs.

**Methods:**

We initially obtained 741 WSIs from the First Affiliated Hospital of Sun Yat-sen University (SYSUFH) for the deep learning model development, optimization, and verification. Additional 318 WSIs from SYSUFH, 212 from Shenzhen People’s Hospital, and 422 from The Cancer Genome Atlas were further collected for multi-centre verification. EfficientNet-B5- and ResNet-50-based deep learning methods were developed and compared using the metrics of recall, precision, F1-score, and areas under the curve (AUCs). A threshold-based tumour-first aggregation approach was proposed and implemented for the label inferencing of WSIs with complex tissue components. Four pathologists of different levels from SYSUFH reviewed all the testing slides blindly, and the diagnosing results were used for quantitative comparisons with the best performing deep learning model.

**Results:**

We developed the first deep learning-based six-type classifier for histopathological WSI classification of lung adenocarcinoma, lung squamous cell carcinoma, small cell lung carcinoma, pulmonary tuberculosis, organizing pneumonia, and normal lung. The EfficientNet-B5-based model outperformed ResNet-50 and was selected as the backbone in the classifier. Tested on 1067 slides from four cohorts of different medical centres, AUCs of 0.970, 0.918, 0.963, and 0.978 were achieved, respectively. The classifier achieved high consistence to the ground truth and attending pathologists with high intraclass correlation coefficients over 0.873.

**Conclusions:**

Multi-cohort testing demonstrated our six-type classifier achieved consistent and comparable performance to experienced pathologists and gained advantages over other existing computational methods. The visualization of prediction heatmap improved the model interpretability intuitively. The classifier with the threshold-based tumour-first label inferencing method exhibited excellent accuracy and feasibility in classifying lung cancers and confused nonneoplastic tissues, indicating that deep learning can resolve complex multi-class tissue classification that conforms to real-world histopathological scenarios.

**Supplementary Information:**

The online version contains supplementary material available at 10.1186/s12916-021-01953-2.

## Background

Lung cancer is the leading killer-cancer worldwide and referred to either non-small cell lung cancer (NSCLC) or small cell lung cancer (SCLC) customarily. Nowadays, with the emerging targeted therapy and immunotherapy, accurate morphological classification is in urgent need [1]. Optical microscopic examination with eyes by pathologists remains the routine in establishing a diagnosis and determining cancer subtypes. However, the scarcity of pathologists and the time-consuming procedure escalate the conflict between clinical demand and actual productivity. Moreover, inter- and intra-observer variations introduce additional bias and risk into histopathology analysis [2, 3]. Fortunately, the digitization of histopathological slides is shifting the way pathologists work and allowing artificial intelligence (AI) to integrate with traditional laboratory workflows.

Over the past few years, deep learning approaches have shown promise in tumour histopathology evaluations [4]. Labour-intensive tasks such as regions of interest (ROIs) detection or segmentation [5, 6], element quantification [7], and visualization [8] can be well executed by deep learning approaches. Experience-dependent problems including histological grading [9], classification or subclassification [10, 11], and prognosis inference [12] have also been solved to some extent with AI approaches. Furthermore, researches on imaging genomics, covering biomarker prediction or discovery [13, 14] and tumour microenvironment (TME) characterization [15] from digital histopathological slides, were explored and demonstrated feasible.

Several deep learning approaches for lung cancer histopathological classification have gained success, in a supervision or weakly supervision manner, via single or multiple convolutional neural network (CNN) models [16–21] (Table 1). Computational tools have been developed for viewing, annotating, and data mining of whole slide images (WSIs) [22–26] (Table 1). Notably, QuPath [22], DeepFocus [23], ConvPath [24], HistQC [25], and ACD Model [26] are referenced in Table 1 as general WSI analysing tools, not specific for lung cancer. Additionally, the relationships between molecular genotypes and morphological phenotypes have been explored in several pioneering studies [16, 17] (Table 1). However, existing advances were confined either to NSCLC, single cohort, or a small number of cases, still a long way to make clinical effects. Furthermore, pulmonary tuberculosis (PTB) cases with nontypical radiographic features require surgical inspections to be differentiated from cancer for potential infectiousness [27]. Organizing pneumonia (OP) is also difficult to be distinguished from bronchogenic carcinoma and thus patients often undergo surgical resection for high suspicion of a malignant tumour [28, 29].

**Table 1:** Glance of deep learning-based lung cancer histological classification algorithms and general slide image analysing tools

Research	Year	Objective	Cohort	AUC	Architecture	Framework	Language
Coudray et al. [16]	2018	Classification between LUAD, LUSC, and NL; mutation prediction (STK11, EGFR, FAT1, SETBP1, KRAS, and TP53)	TCGA (1634 slides); NYU (340 slides)	0.970 (classification) 0.733–0.856 (mutation)	Inception-V3	TensorFlow	Python
Yu et al. [17]	2020	Identification of histological types and gene expression subtypes of NSCLC	ICGC (87 LUAD patients, 38 LUSC patients); TCGA (427 LUAD patients, 457 LUSC patients)	0.726–0.864	AlexNet; GoogLeNet; VGGNet-16; ResNet-50	Caffe	Python
Gertych et al. [18]	2019	Histologic subclassification of LUAD (5 types)	CSMC (50 cases); MIMW (38 cases); TCGA (27 cases)	Accuracy, 0.892 (patch-level)	GoogLeNet; ResNet-50; AlexNet	Caffe	MATLAB
Wei et al. [19]	2019	Histologic subclassification of LUAD (6 types)	DHMC (422 LUAD slides)	0.986 (patch-level)	ResNet-18	PyTorch	Python
Kriegsmann et al. [20]	2020	Classification between LUAD, LUSC, SCLC and NL	80 LUAD, 80 LUSC, 80 SCLC and 30 controls from NCT	1.000 (after strict QC)	Inception-V3	Keras (TensorFlow)	R
Wang et al. [21]	2020	Classification between LUAD, LUSC, SCLC, and NL	SUCC (390 LUAD; 361 LUSC; 120 SCLC; and 68 NL slides); TCGA (250 LUAD and 250 LUSC slides in good quality)	0.856 (for TCGA cohort)	Modified VGG-16	TensorFlow	Python
QuPath [22]	2017	Tumour identification, biomarker evaluation, batch-processing, and scripting	Specimens of 660 stage II/III colon adenocarcinoma patients from NIB	/	/	/	JAVA
DeepFocus [23]	2018	Detection of out-of-focus regions in WSIs	24 slides from OSU	/	CNN	TensorFlow	Python
ConvPath [24]	2019	Cell type classification and TME analysis	TCGA (LUAD); NLST; SPORE; CHCAMS	/	CNN	/	MATLAB; R
HistoQC [25]	2019	Digitization of tissue slides	TCGA (450 slides)	/	/	/	HTML5
ACD model [26]	2015	Colour normalization for H&E-stained WSIs	Camelyon-16 (400 slides); Camelyon-17 (1000 slides); Motic-cervix (47 slides); and Motic-lung (39 slides)	0.914 (for classification)	ACD	TensorFlow	Python

*Abbreviations: LUAD*, lung adenocarcinoma; *LUSC*, lung squamous cell cancer; *NL*, normal lung; *TCGA*, the Cancer Genome Atlas; *NYU*, New York University; *ICGC*, International Cancer Genome Consortium; *CSMC*, Cedars-Sinai Medical Center; *MIMW*, Military Institute of Medicine in Warsaw; *DHMC*, Dartmouth-Hitchcock Medical Center; *NCT*, National Center for Tumor Diseases; *QC*, quality control; *SUCC*, Sun Yat-sen University Cancer Center; *NIB*, Northern Ireland Biobank; *OSU*, Ohio State University; *NLST*, National Lung Screening Trial; *SPORE*, Special Program of Research Excellence; *CHCAMS*, Cancer Hospital of Chinese Academy of Medical Sciences; *H&E*, haematoxylin and eosin; *WSIs*, whole slide images; *ACD*, adaptive colour deconvolution

Here, we developed a deep learning-based six-type classifier for the identification of a wider spectrum of lung lesions, including lung cancer, PTB, and OP. EfficientNet [30] and graphic processing unit (GPU) were utilized for better efficacy. We also implemented a threshold-based tumour-first aggregation method for slide label inferencing, which was inspired by clinical routine and proved to be effective through multi-centre validation. Extended comparison experiments and statistical analyses were conducted for the verification of model efficiency, efficacy, generalization ability, and pathologist-level qualification. We intended to test the hypothesis that deep learning methods can identify lung cancer and mimics histologically with high accuracy and good generalization ability.

## Methods

**Fig. 1:**
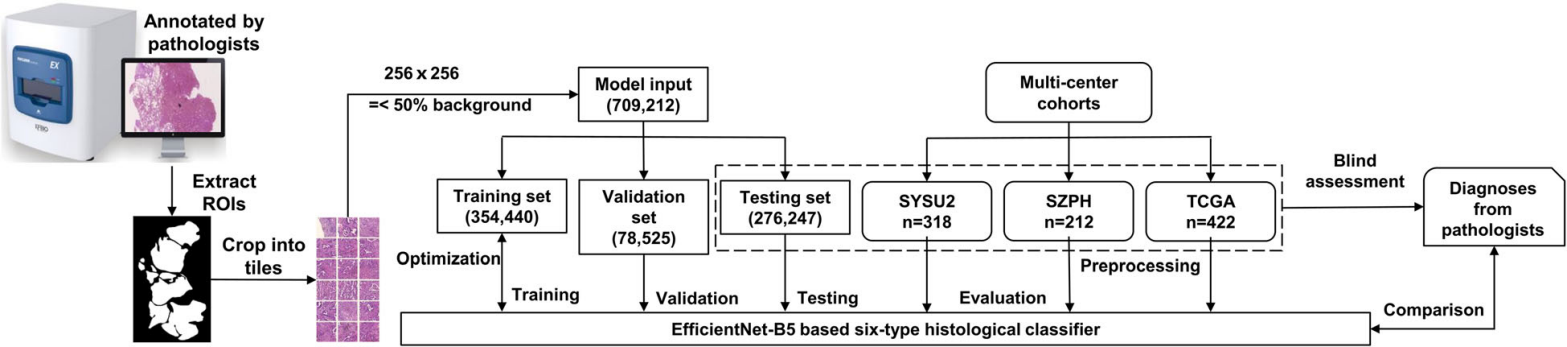
The data analysis workflow in details. ROIs of the H&E-stained slides were extracted by masking on the annotated regions and cropped into 256 × 256 pixels tiles to train the EfficientNet-B5 networks. Tile-level predictions were aggregated to inference the slide-level diagnoses. Tile numbers are in parentheses, and *n* is slide number

The study workflow is illustrated in Fig. 1. First, specimens were scanned and digitized into pyramid-like structured WSIs. Second, WSIs were reviewed and annotated by pathologists. Third, ROIs were extracted and cropped into tiles for model development. Fourth, the deep convolutional neural network was trained and optimized to gain the optimum classification performance. Fifth, tile-level predictions were aggregated into slide-level predictions. Ultimately, multi-centre tests were conducted for adequate validations of the model’s generalization abilities.

### WSI datasets

The initial dataset consisted of 741 haematoxylin and eosin (H&E)-stained lung tissue slides with a confirmed diagnosis of either LUAD, LUSC, SCLC, PTB, OP, or NL from the First Affiliated Hospital of Sun Yat-sen University (SYSUFH) (Table 2). The inclusion criterion was that each slide should show typical lesions indicative of one of the aforementioned diagnostic categories. Before the WSI annotation, two pathologists reviewed all the histological slides of each case microscopically, including immunohistochemistry and histochemical staining slides used for auxiliary diagnosis, and accessed to patients’ medical reports when necessary. Cases with confirmed diagnosis (one slide per case) were included in this study. The slides were then scanned with a KF-PRO-005-EX scanner (KFBIO, Ningbo, China) at × 40 equivalent magnification (0.25 μm per pixel) and digitized into KFB format. In pursuit of an unbiased assessment, the diagnostic annotations were reviewed by pathologists with at least 7 years of clinical experience from the Department of Pathology of SYSUFH according to the 2015 World Health Organization (WHO) classification criteria of lung tumours [1].

**Table 2:** Details of SYSU1 dataset for the development of six-type classifier

Number of slides (tiles)							
Subsets	LUAD	LUSC	SCLC	PTB	OP	NL	SUM
**Training**	210 (179,402)	77 (51,949)	65 (17,342)	43 (22,617)	46 (17,987)	70 (65,143)	511 (354,440)
**Validation**	45 (43,153)	18 (14,552)	16 (1077)	11 (3047)	10 (4170)	15 (12,526)	115 (78,525)
**Testing**	43	16	22	10	10	14	115 (276,247)
**SUM**	298	111	103	64	66	99	741 (709,212)

### Data pre-processing

The raw gigabyte multi-layer WSIs from SYSUFH were converted from KFB to TIFF format with the KFB_Tif_SVS2.0 tool (provided by the scanner vendor KFBIO) for compatibility with mainstream computer vision tools. To retain both global overview and local details, the images of × 20 equivalent magnification (0.5 μm per pixel) was adopted throughout the processing procedure. The TIFF-format WSIs were manually annotated by the pathologists using the ASAP platform [31], with separate areas of coloured irregular polygons responsible for a certain histopathological lung tissue type. Tumorous and inflammatory regions were obtained by masking annotated areas, and normal regions were retrieved by excluding the background of normal lung slides with Otsu’s method [32]. The annotation guaranteed that no non-lesion tissues were included in the annotated area, and thus, some lesion areas that were difficult to be marked clearly may be lost. These outlined areas were annotated with their respective categories, including LUAD, LUSC, SCLC, PTB, and OP. Normal lung slides were derived from normal adjacent tissues of cases with the above diseases. The selected normal lung WSIs referred to the tissues of the whole slides that were normal without any lesions. Specifically, unannotated regions of neoplastic slides were not considered normal due to the rigorous labelling method that excluded minor areas of tumour tissue surrounded by mostly normal tissues. ROIs were traversed and tailored into non-overlapping tiles at the size of 256 × 256 pixels with a sliding window (stride = 256) to match the input scale of CNNs and avoid overfitting. Tiles with over 50% background space were removed to reduce noise and redundancy. The tile distributions are detailed in Table 2.

### Deep neural networks

A CNN with high accuracy and low tuning costs was our aspirational framework. The EfficientNet networks benefited from compound scaling and auto architecture search, achieving state-of-the-art accuracy on ImageNet [33] with fewer floating-point operations per second (FLOPs). PyTorch supported the EfficientNet network up to the B5 version at the time this study was conducted. Hence, EfficientNet-B5 was adopted for the histopathological classification task with its last fully connected layer replaced by a Softmax layer that output a six-dimension vector. To train and optimize the networks, we randomly divided the slides at the slide level into the disjoint training, validation, and testing sets (Table 2). ResNet is another popular CNN architecture that frequently appeared in research articles. Therefore, we also fine-tuned a ResNet-50 network using the same data and settings as EfficientNet-B5 and threw the same testing slides to conduct a fair comparison between the two network models.

### Network training

Limited by the reality of strict privacy policies and nonuniform medical management systems, most medical samples are inaccessible, especially labelled samples [34, 35]. Hence, transfer learning techniques were employed to train the EfficientNet-B5 network given our relatively moderate training dataset. The training process was comprised of two steps. First, we initialized the network with default weights transferred from the ImageNet dataset, froze all the layers except the last fully connected layer, and trained it with our data. Second, we unfroze the frozen layers and finetuned the whole network to fit the target best. The parameters of the trainable layers were modified and optimized referring to the cross-entropy between the predictions and the ground truths. The initial learning rate was 0.0005, and the optimizer was Adam [36], with both momentum and decay set as 0.9. On-the-fly data augmentations, including rotating between 0 and 30°, flipping horizontally or vertically, random brightness or contrast or gamma, zooming in or out, shifting, optical or grid distortion, and elastic transformation, were performed to aggrandize data varieties. Except for horizontal flipping, all the other augmentation operations were conducted with a certain probability, either 0.3 or 0.5. To improve the learning properties on convergence, pixels were rescaled from 0 to 255 to 0–1 by dividing 255, and then *Z*-score-normalized with mean (0.485, 0.456, 0.406) and std. (0.229, 0.224, 0.225). The training process lasted for 60 epochs, and the optimized model with the minimum loss was saved and adopted.

### Whole-slide label inferencing with threshold-based tumour-first aggregation

Outputs of the network were tile-level predictions that should be aggregated into slide-level diagnoses. Traditionally, a tile would be inferred as the class with the maximum prediction probability. Classical aggregation approaches usually fell into two categories to draw the slide-level inference. One is known as the majority voting method, which counts the tile number per class and assigns a slide with the label corresponding to the most numerous class, and the other is the mean pooling method that adds the probabilities of each class and deduces the slide label from the maximum mean class probability. In our datasets, compound tissue components may coexist in one slide. For example, normal, inflammatory, and neoplastic components may scatter across different regions of a tumorous slide; meanwhile in this study, only one major type of neoplastic component would appear in the tumorous slide label. Accordingly, we proposed a two-stage threshold-based tumour-first aggregation method that fused the majority voting and probability threshold strategies. Pathologists often encountered cases in which multiple lesions coexisted, for example lung cancer and PTB or OP may coexist in one H&E slide. If all lesion types were equally treated and the type with the highest prediction probability regarded as the slide-level diagnosis, the model output may miss the cancer lesion due to its small size, which could be much more harmful to patients. Therefore, we aimed to improve the diagnostic sensitivity of cancer and proposed the tumour-first approach. Our method prioritized the tissue types according to the severity of diseases and reported the most threatening tissue type, especially tumorous types.

It is reasonable to set different thresholds for different lesion types. For inflammatory diseases, the threshold range of PTB was initially set slightly lower than that of OP, because PTB is more characteristic morphology microscopically. The threshold range of normal lung tissue was set as high as possible to improve the diagnostic precision. Because LUAD, LUSC, and SCLC are all tumour types, their thresholds should be the same. Also, the thresholds should be roughly inversely proportional to disease severity in order to improve sensitivity. Consequently, the thresholds were set to satisfy the criteria: Tumour_threshold < PTB_threshold < OP_threshold < NL_threshold.

Our expert pathologists agreed the threshold-based tumour-first idea and suggested the threshold ranges according to clinical experiences as following: Tumour = [0.1, 0.5], PTB = [0.2, 0.5], OP = [0.3, 0.5], and NL = [0.7, 0.95]. We adopted these threshold principles and ranges and applied a grid search method with a step of 0.05 to obtain the optimal threshold settings on the first testing dataset SYSU1 (Sun Yat-sen University dataset 1). Accordingly, we got 450 groups of thresholds and calculated their corresponding micro-average and macro-average AUCs. By descending order micro-average AUC first, descending order macro-average AUC as an additional condition, the combination of Tumour (LUAD, LUSC, or SCLC) = 0.1, PTB = 0.3, OP = 0.4, and NL = 0.9 satisfied the principles aforementioned and ranked the top for SYSU1 testing cohort (Additional file 2: Table S1); therefore, it was selected as the threshold setting in the following work.

After the thresholds being defined, the two-stage aggregation was implemented. In the first stage, the aggregation principle was applied to draw each tile’s label and formulated as following (Additional file 1: Figure S1): (i) if the prediction probability of NL exceeded 0.9, the tile was inferred as NL; (ii) otherwise, if the probability of any neoplastic category was greater than 0.1, the label was assigned with the neoplastic class of the maximum probability; (iii) otherwise, if the prediction values of PTB or OP were higher than other thresholds, the corresponding class label was assigned; and (iv) if any of the above conditions were unmet, the tile would be labelled as the class with the maximum probability value. In the second stage, a similar protocol was applied with the tile number per class divided by the total tile number used as the input vector (Additional file 1: Figure S2). We got each tile’s label from the first stage and counted the number of supporting tiles in each class; the number was then divided by the sum of all tiles to obtain the slide-level probability proportion of each class; and finally, we used the slide-level proportion as the input of the second stage to inference the slide-level label. As result, the tile-level predictions aggregated to reach the human-readable slide-level diagnoses in accordance with medical knowledge.

### Multi-centre model testing

To explore the generalization ability of our classifier, further validations were conducted on four independent cohorts, including two inner cohorts SYSU1 and SYSU2 (Sun Yat-sen University dataset 2), and two external cohorts SZPH (Shenzhen People’s Hospital dataset) and TCGA (The Cancer Genome Atlas dataset) (Table 2, Table 3, and Additional file 3: Table S2). Both SYSU1 and SYSU2 datasets came from the First Affiliated Hospital of Sun Yat-sen University. All the slides were anonymized to protect patients’ privacy. Different from and without intersection with the slides subjected to the development of the model, the slides for testing had clinical diagnosis labels only and obtained an inferred diagnosis from the model. Tiles were extracted from the whole slide exhaustedly excluding the background, allowing 10% overlapping with adjacent tiles, and those with tissue proportion less than 20% were filtered for computation efficiency. Appropriate measurements, including recall, precision, F1-score, accuracy, and AUC were computed to quantify and compare the models’ performances across these four testing cohorts.

**Table 3:** Multi-centre cohorts collected for model validation

Cohorts	LUAD	LUSC	SCLC	PTB	OP	NL	SUM
**SYSU2**	56	64	52	30	25	91	318
**SZPH**	60	75	43	0	0	34	212
**TCGA**	141	134	0	0	0	147	422

### Comparison between the deep learning model and stratified pathologists

Four pathologists of different professional level diagnosed the WSIs with ASAP independently and blindly in a single stretch and documented the time they spent. Then, we collected their diagnosis results for performance evaluations and comparisons with our six-type classification model.

### Visualization of the predictions

Heatmap is widely used for visualization due to its variegated colours and expressive exhibitions. In this work, heatmaps were plotted overlying the tiles, displaying equivalent colours corresponding to the tile-level class probability that ranged from 0 to 1. A more saturated colour indicated a larger probability. As appropriate, the coordinate system marked where specific tiles located was omitted for integral aesthetics. Receiver operating characteristic curves (ROCs) were plotted to show the dynamic tendency in which sensitivity varied with specificity. Bar plot and Cleveland graph were plotted to illustrate tile distributions within slides and across cohorts. Sankey figure was drawn to show the comparisons between our deep learning model and the most experienced pathologist.

### Statistical analysis

To evaluate the performances of our model and pathologists, precision, recall, F1-score, AUC, micro-average AUC, and macro-average AUC were calculated in Python with the scikit-learn [37] library using functions including classification_report, auc, and roc_curve. Micro- and macro-AUCs were computed as sample- and class-average AUCs, respectively. 95% CIs were estimated for categorical AUC, micro-average AUC, and macro-average AUC by bootstrapped [38] resampling the samples 10,000 times. The intraclass correlation coefficient (ICCs) were calculated with the ‘irr’ package [39] in R using the ‘oneway’ model, the corresponding 95% CIs were also given by 10,000-fold bootstrapping. ICC ranges from 0 to 1, and a high ICC denotes good consistency. Conventionally, when ICC > 0.75 and *P* < 0.05, high reliability, repeatability, and consistency were indicated [40].

### Hardware and software

The raw WSIs were viewed with K-Viewer (provided by the scanner vendor, KFBIO). OpenSlide [41] (version 1.1.1) and OpenCV [42] (version 4.1.1) in Python (version 3.6.6) were utilized for image extracting and analysing. The main working platform was a high-performance computing node equipped with dual NVIDIA P100 16GB Volta GPUs, and the deep learning model was constructed, trained, and validated with PyTorch [43] (version 1.2.0) on a single GPU. Scikit-learn (version 0.21.2) and Matplotlib [44] (version 2.2.2) in Python undertook major estimation and visualization work cooperatively. The ‘gcookbook’ and ‘tidyverse’ packages in R (version 3.6.1) were adopted to draw bar plots and Cleveland graphs.

## Results

### Internal cohort testing

A total of 741 lung-derived digital WSIs, consisting of 512 tumorous tissues, 130 inflammatory tissues, and 99 normal tissues from the SYSUFH, constituted the initial dataset and were randomly divided into the training (*n* = 511 slides), validation (*n* = 115 slides), and internal testing (SYSU1) (*n* = 115 slides) subsets (Table 2). The WSIs for training and validation were annotated by experienced pathologists and reviewed by the head of the Pathology Department at SYSUFH, and only ROIs were extracted and tessellated into small 256- × 256-pixel tiles at × 20 magnification as inputs of the EfficientNet-B5 network. As for the testing slides, simply diagnostic labels were assigned and the whole excluding background was utilized and pre-processed in the same fashion as annotated slides. In total, 709,212 tiles yielded, of which 432,965 joined the training and validation processes and 276,247 were subject to evaluating the classification performance of the model. The tile distributions are detailed in Table 2. With the training and validation datasets, we developed a deep learning-based six-type classifier that can identify histopathological lung lesions of LUAD, LUSC, SCLC, PTB, OP, and normal lung (NL).

**Fig. 2:**
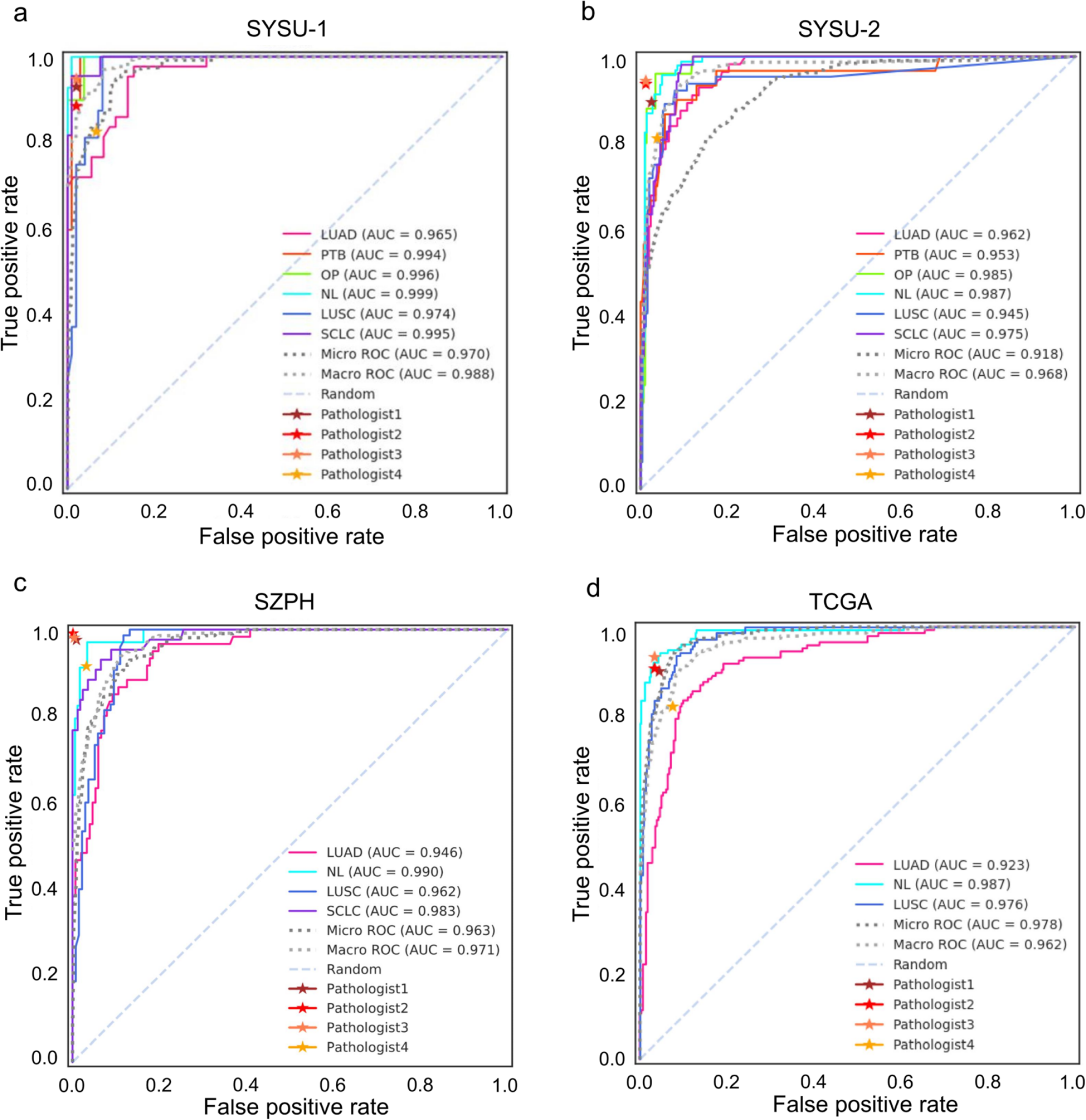
High AUCs achieved across multiple cohorts. AUC was utilized to measure the performance of the model on different testing cohorts, including **a** the subset of the initial cohort SYSU1, **b** an independent internal cohort SYSU2, **c** an external cohort from Shenzhen People’s Hospital (SZPH) that contained 4 types of lung tissues, and **d** a public cohort from ‘TCGA’ which was actually a subset consisting of slides randomly selected from the TCGA-LUAD and TCGA-LUSC projects. Blind tests were conducted on all the cohorts by four pathologists of three levels (Pathologist1 is senior attending, Pathologist2 and Pathologist3 are junior attending, and Pathologist4 is junior); performance of each pathologist on each cohort was depicted as a star in **a**–**d**, respectively

Tested on the internal independent cohort of 115 WSIs, micro- and macro-average AUCs of 0.970 (95% CI, 0.955–0.984) and 0.988 (95% CI, 0.982–0.994) were achieved respectively (Fig. 2a). AUCs for all tissue types were above 0.965, and the successes in SCLC (0.995), PTB (0.994), and OP (0.996) suggested the model competent in distinguishing cancerous and noncancerous lung diseases. Precision, recall, and F1-score were adopted for static assessment (Table 4). It was gratifying that SCLC and noncancerous tissues tended to obtain high precisions, and SCLC, NL, and OP even achieved 1. This meant fewer false positives for SCLC and mild diseases thus lower the risks of serious consequences of missed diagnoses. Meanwhile, cancerous tissues were observed to obtain high recalls, which coincided with the purpose of high sensitivities of malignant tissues. In brief, the deep learning-based six-type classifier exhibited substantial predictive power in the internal independent testing. The whole slide level confusion matrixes (Additional file 1: Figure S3) for each testing cohort illustrated the misclassifications by our method.

**Table 4:** Model performances across SYSU1, SYSU2, SZPH, and TCGA testing sets

Metrics	LUAD	LUSC	SCLC	PTB	OP	NL	Macro-avg
Cohorts							
**Precision**							
**SYSU1**	0.80	0.75	**1.00**	**0.89**	**1.00**	**1.00**	0.91
**SYSU2**	0.85	**0.88**	0.79	0.80	0.88	0.96	0.86
**SZPH**^a^	**0.97**	0.84	0.94	–	–	**1.00**	**0.94**
**TCGA**^b^	0.82	0.70	–	–	–	**1.00**	0.84
**Macro-avg**	0.86	0.79	0.91	0.85	0.94	0.99*	0.89
**Recall**							
**SYSU1**	**1.00**	0.75	0.77	0.80	0.60	0.93	0.81
**SYSU2**	0.84	0.72	**0.94**	**0.93**	**0.84**	**0.95**	**0.87**
**SZPH**^a^	0.93	**0.97**	0.67	–	–	0.91	**0.87**
**TCGA**^b^	0.68	0.94	–	–	–	0.78	0.80
**Macro-avg**	0.86	0.85	0.79	0.87	0.72	0.89*	0.84
**F1-score**							
**SYSU1**	0.89	0.75	**0.87**	0.84	0.75	**0.96**	0.84
**SYSU2**	0.85	0.79	0.86	**0.86**	**0.86**	0.95	0.86
**SZPH**^a^	**0.95**	**0.90**	0.78	–	–	0.95	**0.90**
**TCGA**^b^	0.74	0.80	–	–	–	0.88	0.80
**Macro-avg**	0.86	0.81	0.84	0.85	0.81	0.94*	0.85

^a^For the SZPH dataset, no PTB or OP WSIs were available

^b^For TCGA dataset, only LUAD, LUSC, and NL WSIs were available

*Maximum Macro-avg value across the datasets of different diseases

Bold font: Maximum value of specific metrics across different data cohorts

### Multi-cohort testing

Another batch of specimens from SYSUFH (SYSU2) (*n* = 318 slides), an external validation dataset from Shenzhen People’s Hospital (SZPH) (*n* = 212 slides), and a randomly selected subset of The Cancer Genome Atlas (TCGA) (*n* = 422 slides) were collected for further multi-cohort testing (Table 3 and Additional file 3: Table S2). Notably, due to the limitation of the external data resource, data for PTB and OP were unavailable for SZPH and TCGA, and data for SCLC was unavailable for TCGA as well. Similarly, AUC, precision, recall, and F1-score were computed for the evaluation of classification performance (Table 4).

Our classifier attained micro-average AUCs of 0.918 (95% CI, 0.897–0.937) (Fig. 2b) and 0.963 (95% CI, 0.949–0.975) (Fig. 2c) for SYSU2 and SZPH, respectively, showing consistent performances in dealing with data from different medical centres. For the public available TCGA subset, the micro-average AUC was 0.978 (95% CI, 0.971–0.983) (Fig. 2d), which surpassed those obtained from both internal and external cohorts. In terms of precision, recall, and F1-score (Table 4), the model performed best with SZPH dataset, followed by SYSU2, and NL was the most accurately distinguished tissue type with macro-average F1-scores of 0.94 across the four cohorts, followed by LUAD with a macro-average F1-score of 0.86. The inherent nature of TCGA and SZPH had limited corresponding experiments to partial categories of lung lesions in this study; meanwhile, the results demonstrated our method’s robustness and insensitivity to the influence of class imbalance. Overall, the histopathological six-type classifier delivered consistent answers to multi-cohort testing, and its flexibility of data bias and applicability of a wider scale bridged the distance between artificial intelligence and actual clinical use. It was reasonable to believe that the model held promise to relieve workloads of pathologists and cover more extensive clinical scenarios.

### Comparison between EfficientNet-B5 and ResNet-50

Table 5 illustrates that ResNet-50 performed comparably with EfficientNet-B5 on the SYSU1 cohort, slightly less accurate on SYSU2. However, EfficientNet-B5 exerted obvious advantages on SZPH and TCGA cohorts. ResNet-50 was competent in common tasks, but inferior in generalization as shallower networks are naturally weaker in learning abstract features which may be crucial for distinguishing slides of multiple sources. Hence, EfficientNet-B5 outperformed ResNet-50 in multi-cohort testing and was selected as the backbone model.

**Table 5:** EfficientNet-B5 outperformed ResNet-50 across four testing cohorts

Cohort	Model	Micro-AUC	Macro-AUC	Accuracy	Weighted-F1-score
**SYSU1**	ResNet-50	0.966	0.985	0.860	0.860
	EfficientNet-B5	0.970	0.988	0.860	0.860
**SYSU2**	ResNet-50	0.887	0.953	0.780	0.770
	EfficientNet-B5	0.918	0.968	0.870	0.870
**SZPH**	ResNet-50	0.713	0.733	0.540	0.520
	EfficientNet-B5	0.963	0.971	0.890	0.900
**TCGA**	ResNet-50	0.967	0.973	0.690	0.680
	EfficientNet-B5	0.978	0.962	0.800	0.810

### Visualizing predictions with heatmaps

**Fig. 3:**
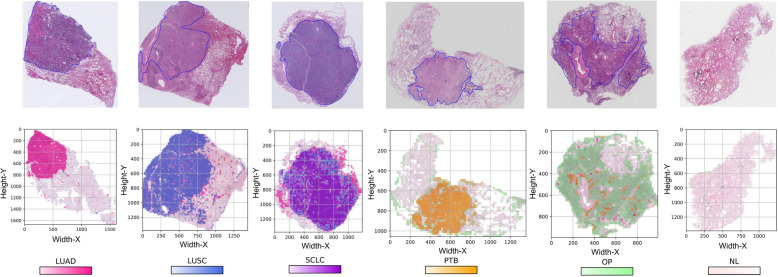
Visualization heatmaps of tissue predictions of LUAD, LUSC, SCLC, PTB, OP, and NL from left to the right, respectively. The top row shows the raw slides with closed blue curves delineating the ROIs annotated by expert pathologists, and the bottom row illustrates the corresponding resulting heatmaps

To see the landscape of whole slide level predictions, heatmaps were plotted as overlays on the tiles with various colours standing for the predicted tissue types. One representative of each tissue type was randomly selected and is visualized in Fig. 3. The first row displayed the WSIs with ROI annotations, and the second row illustrated the resulting probability heatmaps paired with the first row. From left to right were the sample heatmaps for LUAD, LUSC, SCLC, PTB, OP, and NL, respectively. In Fig. 3, the predictions of tiles and subregions were clearly observed and mapped to the in situ tissues. The whole slide landscapes of predictions were generally a mix of tissue components, among which the predominant component of the same priority contributed more to the final diagnostic conclusion. Figure 3 also illustrates that the suggested regions by our six-type classifier were highly consistent with the ROIs annotated by the pathologists. For example, the highlighted regions of SCLC, PTB, and OP heatmaps were perfectly matched to their corresponding ROI annotations in the upper row, and the predicted region of LUAD coincided with the main ROI though missed about 30% of the actual lesions. Notably, cancerous components merely appeared in noncancerous slides, and the prominent components tended to present like a gobbet. In addition, the margins of noncancerous slides seemed to be predicted as OP. We also generated the heatmaps (Additional file 1: Figure S4) to present the false-positive prediction cases. In these false-positive cases, cancer cases were predicted as other types of cancer, and NL cases were predicted as PTB or OP cases. In brief, the heatmaps allow to overview predictions of the whole slides intuitively, discover the underlying histopathological patterns, and simplify the result interpretations.

### Contesting with pathologists

To compare our model with pathologists for the diagnosis of lung lesions, four pathologists from three different training levels (senior attending, junior attending, and junior) were invited to independently and blindly review all the H&E-stained slides from four testing cohorts by manual inspection alone. True-positive rate (TPR) and false-positive rate (FPR) were calculated for each pathologist and attached to the ROCs as different coloured five-pointed stars (Fig. 2). We can see the NL curves (cyan) over some stars, and LUAD curves (hot pink) under or close to the stars in most cases. Pathologist3 reached the top rank in SYSU1, SYSU2, and TCGA, albeit at junior attending status. Disparities between attending pathologists existed but not made much difference. Roughly, our model accomplished comparable performance with pathologists, and even better in some cases.

Aiming at quantifying the performance consistency, ICCs under 95% CIs among the pathologists and our model were calculated. As listed in Table 6, our method achieved the highest ICC of 0.946 (95% CI, 0.715, 0.992) with the ground truth in TCGA, 0.945 (95% CI, 0.709, 0.992) with Pathologist3 in SYSU1, 0.960 (95% CI, 0.783, 0.994) with Pathologist2 in SYSU2, and 0.928 (95% CI, 0.460, 0.995) with Pathologist3 in SZPH, respectively. All the ICCs were above 0.75 (*P* < 0.05), and the model behaved closest to Pathologist3 overall, who was the best performing pathologist in point of blind inspection on the four cohorts.

**Table 6:** High ICCs between the model and pathologists across four independent testing cohorts indicate high consistency and comparable performance

Raters	Six-type classification model (ICC^a^ with 95% CI^b^)			
	SYSU1	SYSU2	SZPH	TCGA
Ground truth	0.941(0.691, 0.991)	0.959 (0.776, 0.994)	0.927 (0.453, 0.995)	**0.946 (0.715, 0.992)**
Pathologist1+++^c^	0.938 (0.677, 0.991)	0.957 (0.767, 0.994)	0.878 (0.215, 0.991)	0.918 (0.592, 0.988)
Pathologist2++^c^	0.873 (0.422, 0.981)	**0.960 (0.783, 0.994)**	0.909 (0.356, 0.994)	0.928 (0.633, 0.989)
Pathologist3++^c^	**0.945 (0.709, 0.992)**	0.945 (0.709, 0.992)	**0.928 (0.460, 0.995)**	0.922 (0.608, 0.988)
Pathologist4+^c^	0.944 (0.707, 0.992)	0.800 (0.200, 0.969)	0.905 (0.538, 0.986)	0.754 (0.086, 0.961)
*P* value^d^	< 0.05	< 0.05	< 0.05	< 0.05

^a^ICCs were computed with the ‘irr’ package for R v3.6.1 using the ‘oneway’ model to measure the reliability and consistency of diagnoses among raters

^b^CIs were given by bootstrapping the samples 10,000 times

^c^‘+’ symbols indicate the levels of pathologists, + means junior, ++ means junior attending, and +++ means senior attending

^d^ICC ranges from 0 to 1, and a high ICC suggests a good consistency. Conventionally, when ICC > 0.75 and *P* < 0.05, high reliability, repeatability, and consistency were indicated

**Fig. 4:**
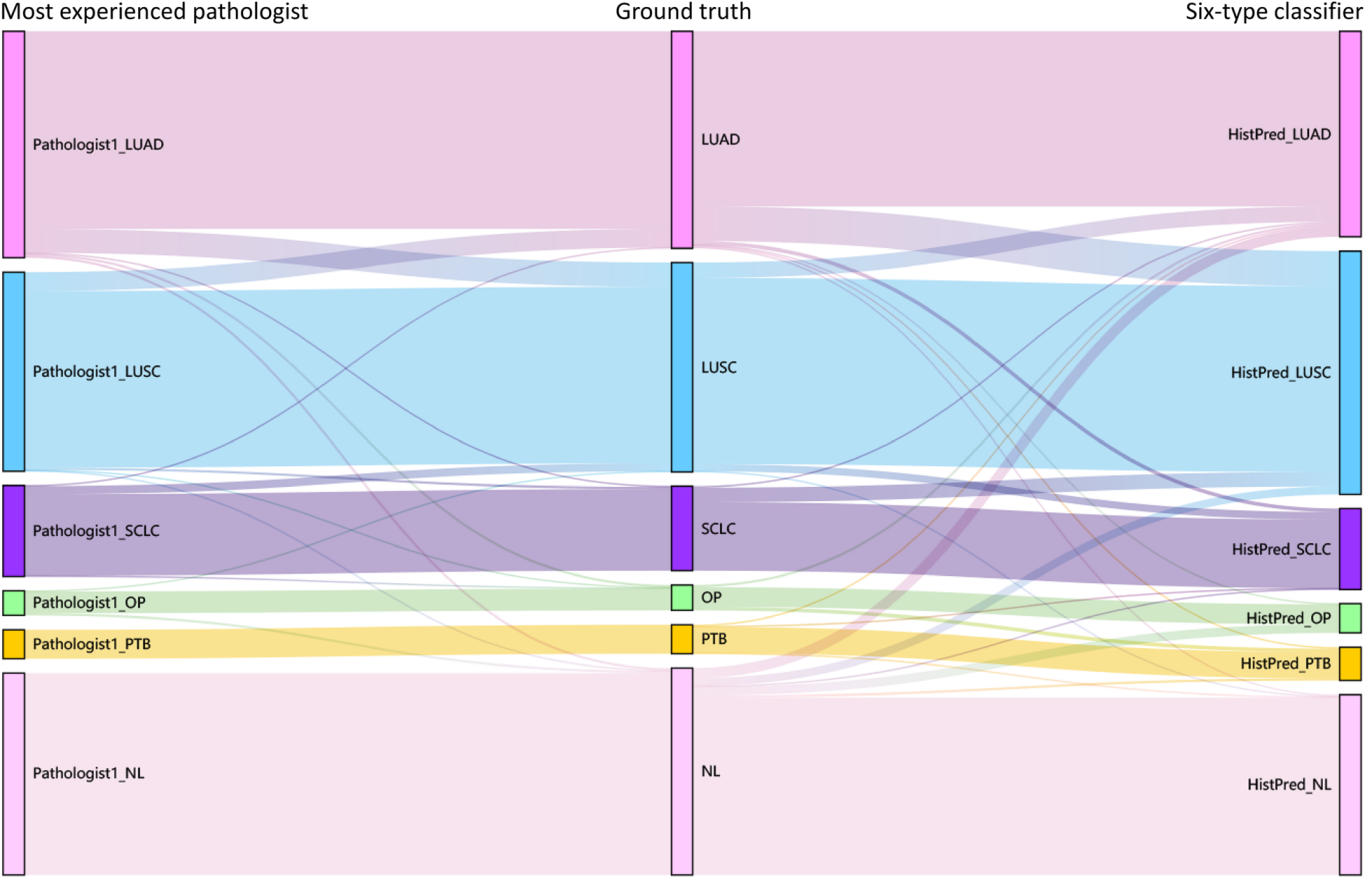
Sankey diagram illustrates the difference among ground truth, best pathologist, and our six-type classifier. From left to right are the predictions by the best pathologist, the ground truth, and the prediction by our six-type classifier

For further insight into the relationships of the resulting predictions, Sankey diagram (Fig. 4) was built to illustrate the difference among the ground truth, the most experienced pathologist (Pathologist1 in Table 6), and our six-type classifier. Taking the ground truth (the middle column) as the benchmark, the spanning curves on the left and right indicate misjudgements of Pathologist1 and our classifier, respectively. The model’s overall performance was comparable with the pathologist and highly consistent with the ground truths. Comparatively, our model made relatively fewer mistakes for LUSC, though more mistakes for LUAD and SCLC. Further, the model was so tumour-sensitive that it tended to gain false positives by predicting NL as suspicious lesions, whereas expert pathologist had much more confident to confirm a disease-free tissue. Table 7 summarizes the cases that were misjudged by at least one pathologist, and over half of the mistakes were corrected by the model. Therefore, our model achieved excellent performance comparable to those of experienced pathologists.

**Table 7:** Misjudges from pathologists were corrected by the six-type classifier

Cohorts	SYSU1	SYSU2	SZPH	TCGA
**Error(s)**^a^	31	84	21	120
**Correction(s)**^b^	22	59	18	90

^a^Errors denote the number of slides misjudged by at least one of the pathologists

^b^Corrections denote the number of those misjudged slides corrected by our six-type classifier

Obviously, manual inspection is labour insensitive. For example, the TCGA cohort cost a pathologist 6 to 10 h to complete a full inspection, while the entire analysis can be done within approximately an hour by the model. Additionally, inter-rater and intra-rater variabilities of manual inspection influenced the final consensus. Compared with pathologist’s manual inspection, our six-type classifier approach is a more stable and cost-effective choice.

## Discussion

Histopathological evaluation has until now been the cornerstone of final cancer diagnosis, directing further examination, treatment, and prognosis. The transition from glass slides under an optical microscope to virtual slides viewed by computers enabled the automatization of inspection and quantitative assessment. Medical AI is demonstrated favourable for improving healthcare qualities and lessening the inequality between urban and rural health services [45]. Lung cancer is threatening millions of lives every year. Though important discoveries have been made during recent years, accurate histopathological classification remains challenging in clinical practice. Certainly, distinguishing LUAD from LUSC is necessary; however, SCLC deserves more attention for its high malignancy and poor survival rate [46]. In addition, tumour generally appears as a mixture of neoplastic and inflammatory lesions, and extensive inflammatory lesions may shield local tumour changes thus contributing to false-negative diagnosis. On the contrary, mistaking nonneoplastic tissues as neoplastic tissues gave rise to the risk of overdiagnosis and overtreatment. Therefore, in order to tackle real clinical problems, we designed the six-type classifier for wider coverage of lung diseases, including lung cancers as well as inflammatory lung diseases.

The histological assessment of lesions involves different staining techniques to make a final diagnosis. In all histological diagnoses, H&E staining must be first and indispensable. In the routine diagnostic procedure for clinicopathological work, pathologists firstly evaluate lesion with benign or malignant using H&E-stained sections. If the lesion is suspected of malignancy, subsequent typing will be conducted using complex immunohistochemistry or molecular detection. If it is benign, especially suspected with inflammatory lesions, such as evaluation of infection with mycobacteria, Ziehl-Neelsen (ZN) staining is needed to confirm the diagnosis. It is true that H&E staining cannot directly identify the pathogens such as mycobacterium; however, lung tissue infected with mycobacterium results in characteristic histological changes, including the granulomas formation which consists of epithelioid macrophages and multinucleated giant cells, often with caseous necrosis centrally. Therefore, we believe that morphology is the first step to recognize disease microscopically. Based on morphological characteristics, our model performed the task of the six-type classification for diagnostic purpose using the H&E-stained tissue.

Our six-type classifier was compatible to other relevant state-of-the-art tools (Table 1) and gained advantages in dealing with complex application scenarios. For example, DeepPath [16] accomplished micro- and macro-average AUCs of 0.970 (95% CI, 0.950–0.986) and 0.976 (95% CI, 0.949–0.993) respectively for the classification of NSCLC, which were not significantly different to ours. Notably, our model performed better in distinguishing NL (0.999 versus 0.984) and LUSC (0.974 versus 0.966), and comparable in LUAD (0.965 versus 0.969 for LUAD). Yu et al. [17] also implemented multiple network architectures to subclassify NSCLC using the TCGA data and achieved an AUC of 0.864, which was 0.114 lower than our TCGA result. Moreover, Kriegsmann et al. [20] adopted Inception-V3 to classify LUAD, LUSC, SCLC, and NL, accomplishing an AUC of 1.000; however, that was achieved after strict quality controls in their data pre-processing phase. Furthermore, Wang et al. [21] conducted a similar classification task without PTB and OP using different feature aggregation methods and compared their efficiencies. Their CNN-AvgFea-Norm3-based RF method achieved an AUC of 0.856 and an accuracy of 0.820 on the TCGA dataset, which was 0.122 loss in AUC and 0.020 gain in accuracy compared with our classifier. Notably, the input dataset in Wang’s study was manually picked up from TCGA and only composed of LUAD (*n* = 250 slides) and LUSC (*n* = 250 slides). These suggested that our classifier could adapt to more complicated situations in real clinical scenarios.

Moreover, we overcame some challenges in data pre- and post-processing. The first challenge was to reduce the class-imbalance of the initial dataset, which needed proper separation at slide- and tile-level. The ROIs varied in size and a slide can have different numbers of ROIs. Hence, we divided the slides into training, validation, and testing sets according to the ROI areas per slide per class, roughly following a ratio of 4:1:1. Nevertheless, some tiles were filtered for low tissue coverage before model training. We examined the distribution of ROI areas by counting the number of tiles per slide (Additional file 1: Figure S5). The general trend in the distribution was that the slide number declined with the tile number increased in both training and validation sets. A majority of the slides got ROIs within 2000 tiles, and the largest tile number was no more than 4000, which suggested cautious annotation strategies and a low chance of excessive presentation of a certain slide, thereby avoiding overfitting in the model training phase to some extent.

Then came the challenge of the aggregation from tile-level prediction to slide-level inference. Note that multiple tissue components usually coexist in a slide. Therefore, the slide-level label should not be determined only based on the tissue type with the most supporting tiles, and tumorous class should be reported first even with fewer cancerous tiles. Most recently, scientists experimented to append heuristic algorithms (e.g., logistic regression, random forest, and support vector machine) which input features based on the tile probability scores generated by CNNs [47, 48]. Campanella et al. applied a random forest algorithm for selecting top suspicious tiles and then trained an RNN model to draw slide-level predictions [49]. However, the feature engineering and extra optimization procedures complicated the classification work and introduced uncertainty to some degrees. In this study, we preferred to testify if a more convenient AI solution could accommodate to clinical use. Accordingly, a set of thresholds advised by expert pathologists conforming to clinical experience was defined and integrated with the majority voting method for the slide-level label inference. Validated on both the inner and inter testing datasets, the thresholds were proved feasible and effective.

Ultimately, we tried to interpret the differences in prediction effectiveness observed in the multi-centre validation experiments. First, we checked and compared the distributions of ROIs across testing cohorts (Additional file 1: Figure S5). Although a similar pattern of tile agglomeration in the testing slides, quite a few slides fell into the interval of 0–500, especially in the SYSU2 and SYSU1 cohorts. The tile distribution of misjudged slides was plotted as a Cleveland graph grouped by cohort (Additional file 1: Figure S6). Not surprisingly, errors occurred intensively in the slides with a tile number less than 500. This happened because small slides were most susceptible to individual tile errors. A closer inspection of the testing set of SYSU1 showed approximate 24.3% of the slides were small specimens, which may partially explain the relatively lower AUCs in SYSU1. SYSU2 cohort was collected due to the substantial number of small sample slides and the imbalance of SYSU1 testing cohort. As a result, the model obtained an improved performance on SYSU2. SZPH cohort was best predicted, which may lie on a relatively even distribution of tiles. When reviewing the TCGA slides, we found some obvious artefacts such as pen marks, margin overlap, and defocus. In addition, staining differences were observed between TCGA and the other three cohorts, which also contributed to the performance diversities.

## Conclusions

The efforts presented in our work highlighted the possibility of predicting a wider spectrum of confusing lung diseases from WSIs using a deep learning model coupled with threshold-based tumour-first aggregation method. With the broad coverage of lung diseases, the rigorous validations on multi-centre cohorts, the improved interpretability of the model, and the comparable consistency with experienced pathologists, our classifier exhibited excellent accuracy, robustness, efficiency, and practicability as a promising assistant protocol, which was close to the complex clinical pathology scenarios.

## Supplementary Information

**Additional file 1: Figure S1.** The workflow diagram for tile-level inferencing. **Figure S2.** The workflow diagram for slide-level inferencing. **Figure S3.** Confusion matrices for testing cohorts of SYSU1, SYSU2, SZPH and TCGA. Rows are the true labels, and columns are the predicted labels. Values in red on the diagonal represent true positive rates (TPRs) or sensitivity, and values elsewhere represent false negative rates (FNRs). A darker square indicates a larger TPR for its corresponding tissue type. **Figure S4.** Heatmaps for representative false positives of each tissue class. The first row shows the raw slides of SCLC, LUAD, LUSC, NL, NL, and PTB, respectively, and the second row corresponds to the prediction heatmaps and the labels inferenced. **Figure S5.** Bar charts displaying the relationship between tile number and slide number. From left to right are bar charts for the training set, validation set, and testing cohorts, respectively. The horizontal axis represents the number of tiles from the same slide, and the vertical axis represents the corresponding slide number. Each colour bar stands for a specific tissue type as the legend shows. **Figure S6.** Cleveland graph showing the tile distribution of model errors. The horizontal axis represents the tile number within a slide, and the vertical axis represents the slide names which are omitted for visual cleanliness. Top to bottom are Cleveland graphs grouped by cohort. Cohort is described by its colour.

**Additional file 2: Table S1.** Grid search report for the threshold optimization in the threshold-based tumour-first aggregation method.

**Additional file 3: Table S2.** TCGA image data of 422 lung samples used in this study.

## Data Availability

The TCGA dataset were derived from the NIH BioProject (TCGA-LUAD and TCGA-LUSC) and available through the GDC Data Portal website (https://portal.gdc.cancer.gov/projects/TCGA-LUAD; https://portal.gdc.cancer.gov/projects/TCGA-LUSC). All other data generated from this study are available upon request to the corresponding author.

## Abbreviations

AI: Artificial intelligence
AUC: Area under the curve
CI: Confidence interval
CNN: Convolutional neural network
FLOP: Floating-point operation per second
FNR: False-negative rate
FPR: False-positive rate
GPU: Graphic processing unit
H&E: Haematoxylin and eosin
ICC: Intraclass correlation coefficient
LUAD: Lung adenocarcinoma
LUSC: Lung squamous cell carcinoma
NL: Normal lung
NSCLC: Non-small cell lung carcinoma
OP: Organizing pneumonia
PTB: Pulmonary tuberculosis
ROC: Receiver operating characteristic curve
ROI: Region of interest
SCLC: Small cell lung carcinoma
SYSU1: Sun Yat-sen University dataset 1
SYSU2: Sun Yat-sen University dataset 2
SYSUFH: First Affiliated Hospital of Sun Yat-sen University
SZPH: Shenzhen People’s Hospital dataset
TCGA: The Cancer Genome Atlas dataset
TME: Tumour microenvironment
TPR: True-positive rate
WHO: World Health Organization
WSI: Whole slide image
