# Supplementary material for: Deep learning-based six-type classifier for lung cancer and mimics from histopathological whole slide images: a retrospective study
Source: BMC Med. 2021 Mar 29;19:80. doi: 10.1186/s12916-021-01953-2 (PMC8006383; doi:10.1186/s12916-021-01953-2)
Supplement: Supplementary file 1 — Additional file 1: Figure S1. The workflow diagram for tile-level inferencing. Figure S2. The workflow diagram for slide-level inferencing. Figure S3. Confusion matrices for testing cohorts of SYSU1, SYSU2, SZPH and TCGA. Rows are the true labels, and columns are the predicted labels. Values in red on the diagonal represent true positive rates (TPRs) or sensitivity, and values elsewhere represent false negative rates (FNRs). A darker square indicates a larger TPR for its corresponding tissue type. Figure S4. Heatmaps for representative false positives of each tissue class. The first row shows the raw slides of SCLC, LUAD, LUSC, NL, NL, and PTB, respectively, and the second row corresponds to the prediction heatmaps and the labels inferenced. Figure S5. Bar charts displaying the relationship between tile number and slide number. From left to right are bar charts for the training set, validation set, and testing cohorts, respectively. The horizontal axis represents the number of tiles from the same slide, and the vertical axis represents the corresponding slide number. Each colour bar stands for a specific tissue type as the legend shows. Figure S6. Cleveland graph showing the tile distribution of model errors. The horizontal axis represents the tile number within a slide, and the vertical axis represents the slide names which are omitted for visual cleanliness. Top to bottom are Cleveland graphs grouped by cohort. Cohort is described by its colour. [file 12916_2021_1953_MOESM1_ESM.docx]

**Supplementary Information - Figures**

**Figure S1.** The workflow diagram for tile-level inferencing

**
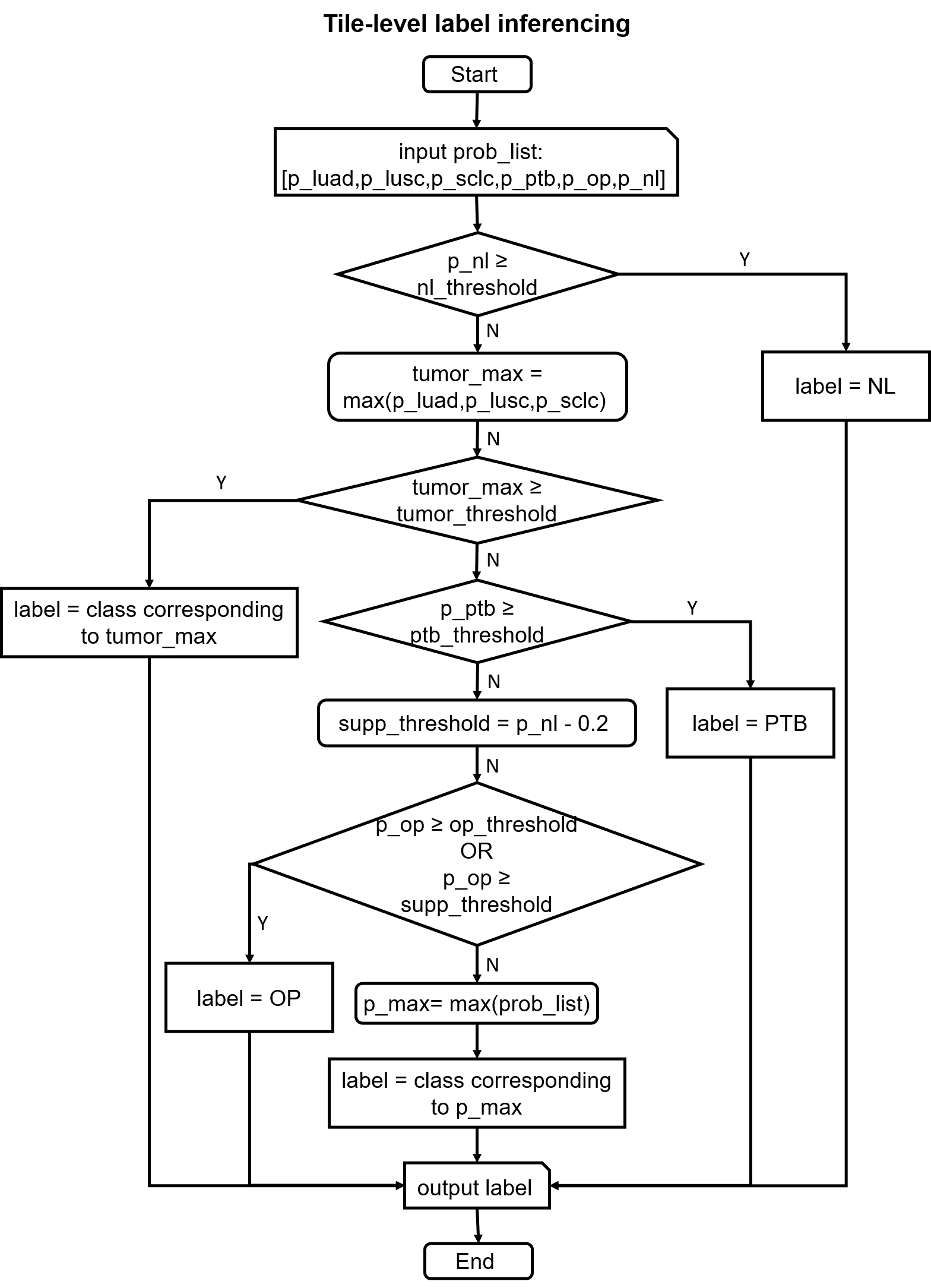
**

**Figure S2.** The workflow diagram for slide-level inferencing.

**
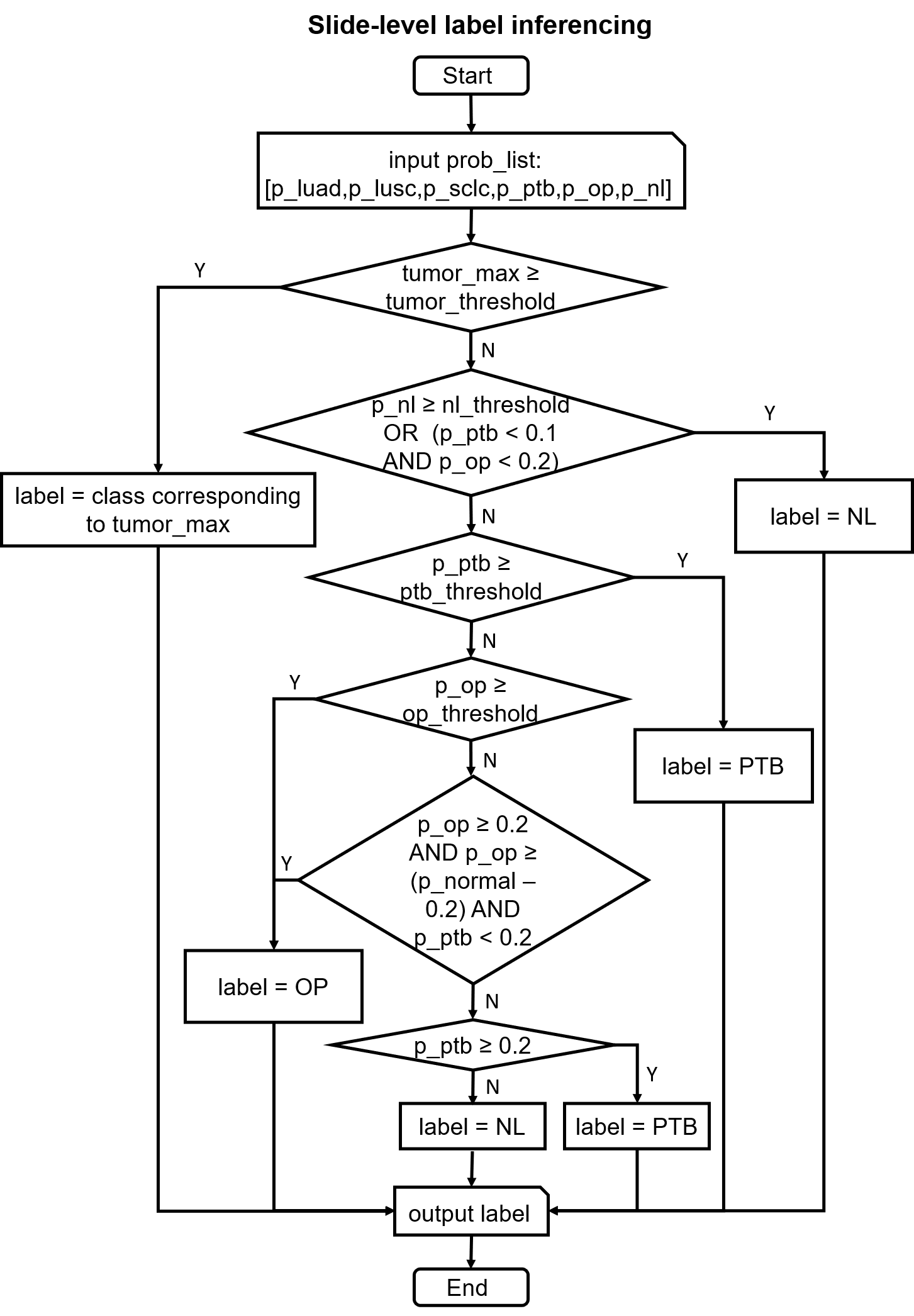
**

**Figure S3. Confusion matrices for testing cohorts of SYSU1, SYSU2, SZPH and TCGA.** Rows are the true labels, and columns are the predicted labels. Values in red on the diagonal represent true positive rates (TPRs) or sensitivity, and values elsewhere represent false negative rates (FNRs). A darker square indicates a larger TPR for its corresponding tissue type.

**
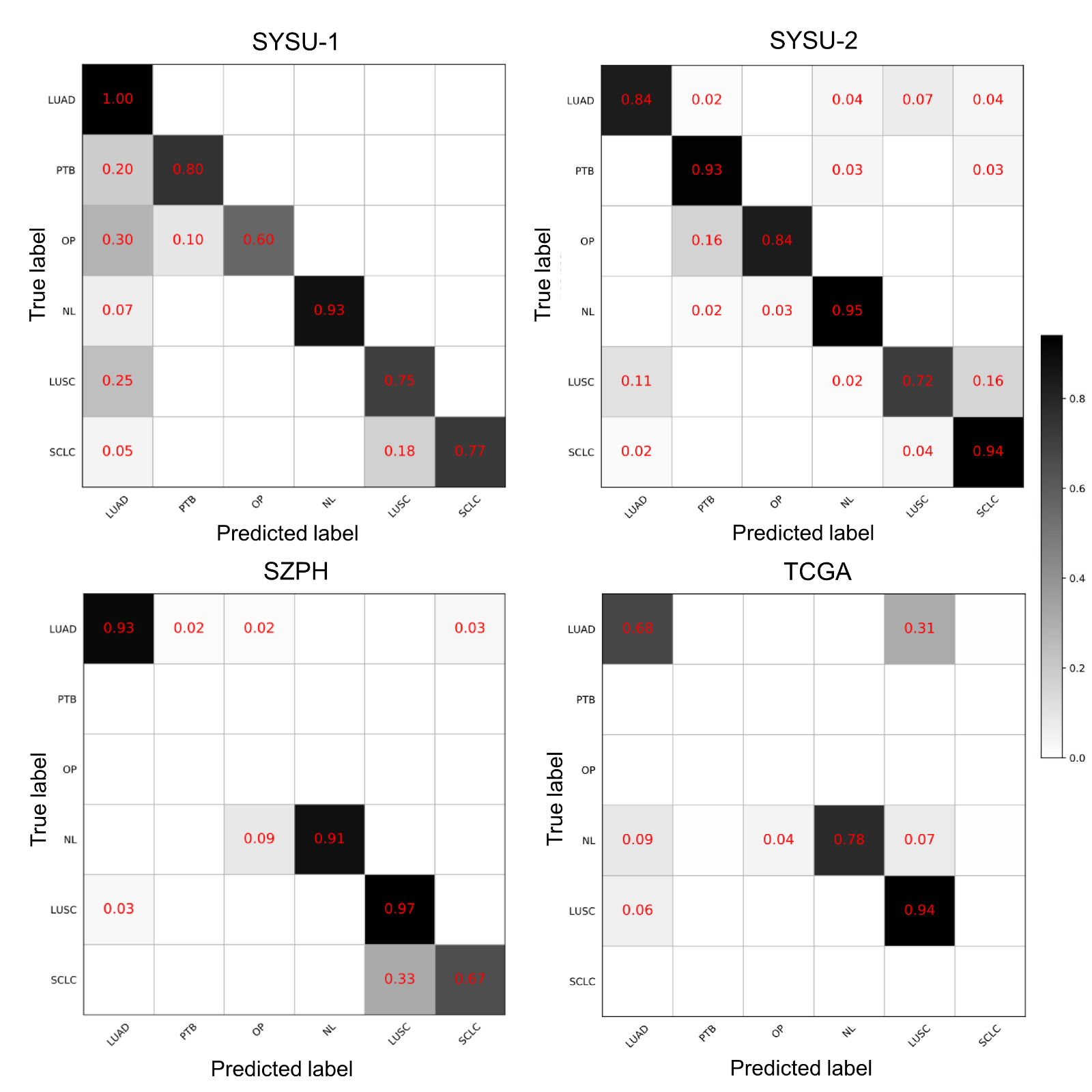
**

**Figure S4. Heatmaps for representative false positives of each tissue class.** The first row shows the raw slides of SCLC, LUAD, LUSC, NL, NL, and PTB, respectively, and the second row corresponds to the prediction heatmaps and the labels inferenced.

**
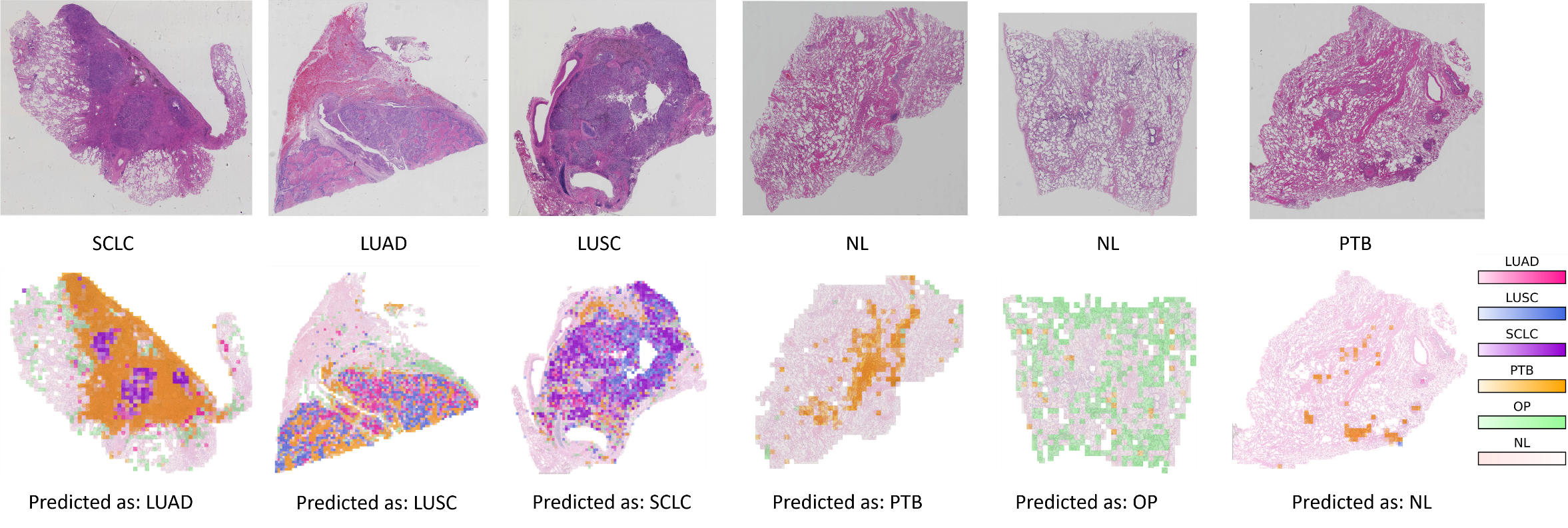
**

**Figure S5. Bar charts displaying the relationship between tile number and slide number.** From left to right are bar charts for the training set, validation set and testing cohorts, respectively. The horizontal axis represents the number of tiles from the same slide, and the vertical axis represents the corresponding slide number. Each colour bar stands for a specific tissue type as the legend shows.

**
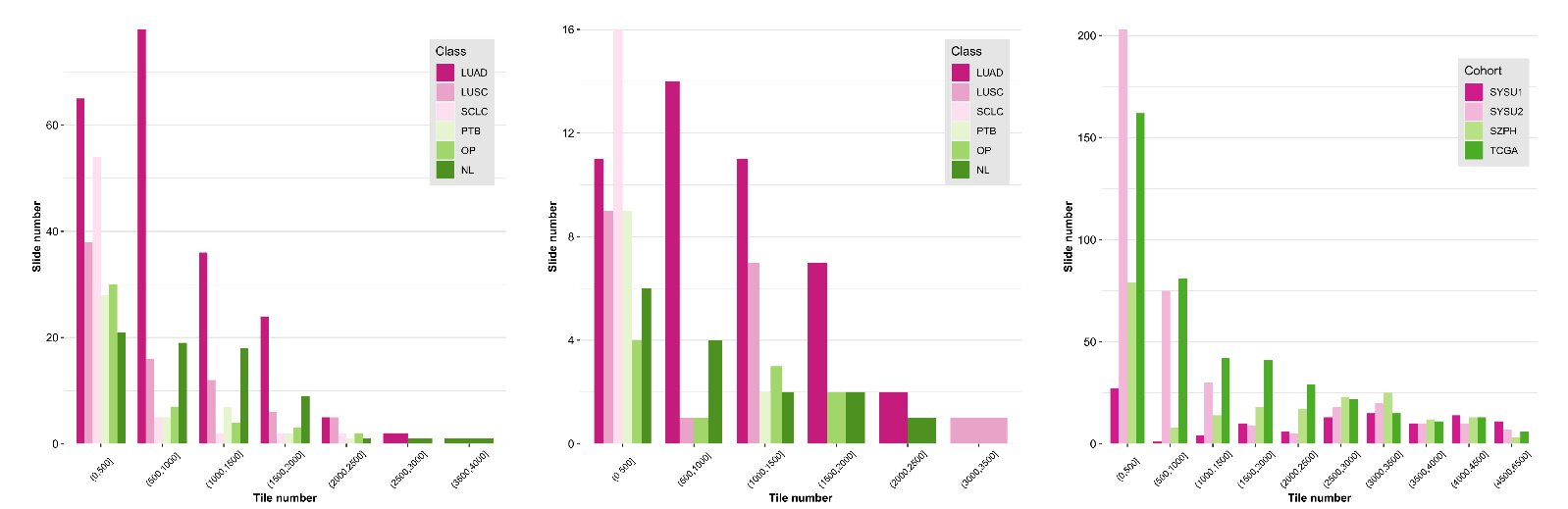
**

**Figure S6. Cleveland graph showing the tile distribution of model errors.** The horizontal axis represents the tile number within a slide, and the vertical axis represents the slide names which are omitted for visual cleanliness. Top to bottom are Cleveland graphs grouped by cohort. Cohort is described by its colour.

**
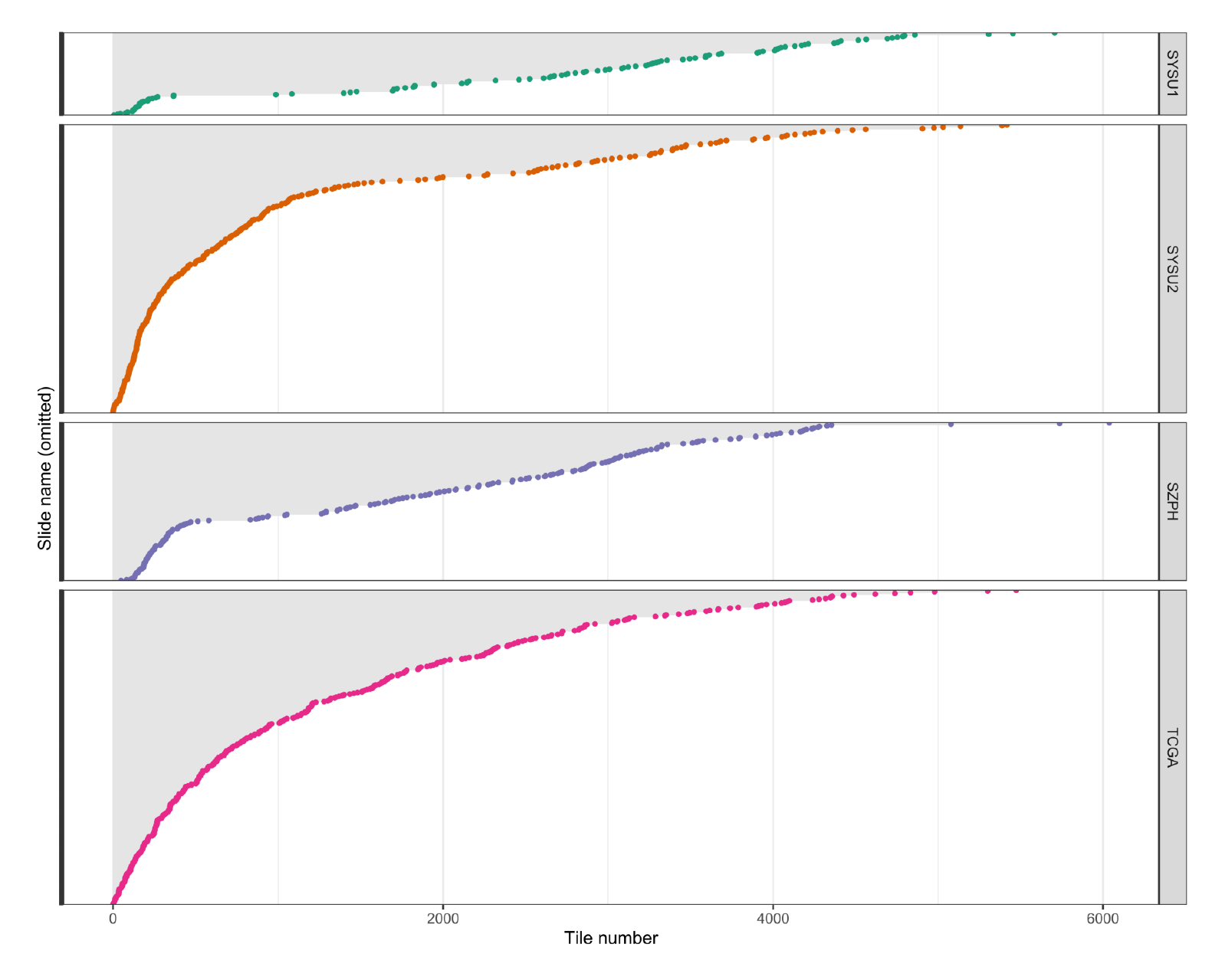
**
